# Heavy Metal Contamination in Adaptogenic Herbal Dietary Supplements: Experimental, Assessment and Regulatory Safety Perspectives

**DOI:** 10.3390/biology14111479

**Published:** 2025-10-23

**Authors:** Agata Jasińska-Balwierz, Patrycja Krypel, Paweł Świsłowski, Małgorzata Rajfur, Radosław Balwierz, Wioletta Ochędzan-Siodłak

**Affiliations:** 1 Department of Pharmacology, Academy of Silesia, 40-555 Katowice, Poland; 2 Institute of Chemistry, University of Opole, Oleska 48 St., 45-052 Opole, Poland; patrycja.krypel1@wp.pl (P.K.); wioletta.siodlak@uni.opole.pl (W.O.-S.); 3 Institute of Biology, University of Opole, Kominka 6, 6a St., 45-032 Opole, Poland; pawel.swislowski@uni.opole.pl (P.Ś.); rajfur@uni.opole.pl (M.R.)

**Keywords:** adaptogens, dietary supplements, heavy metals contamination, regulatory safety, consumer safety

## Abstract

**Simple Summary:**

Herbal dietary supplements with adaptogens, such as ginseng, ashwagandha, rhodiola, and schisandra, are popular worldwide because they are believed to reduce stress and support health. However, plants can absorb harmful metals like lead and nickel from soil, water, and air. These metals can damage the brain, heart, liver, and other organs even at low levels. In our study, we tested eleven commonly available adaptogenic supplements sold in Poland. Using laboratory methods, we measured the levels of several metals. We found that most of the products contained too much lead and nickel, sometimes more than three times above safe limits. Tablets were the most contaminated, which may be linked to processing during production. The highest levels of contamination were found in schisandra products, and supplements made from Indian raw materials often had more nickel than those from China. These findings show that many adaptogenic supplements do not meet safety standards and may pose health risks, especially if used regularly. Stronger control of dietary supplements and better regulations are needed to protect consumers. Choosing herbal medicines, which must follow stricter safety rules, could be a safer alternative for people seeking the benefits of adaptogens.

**Abstract:**

While adaptogens are popular in dietary supplements for their health-promoting properties, their safety is compromised by the risk of heavy metal contamination, a threat amplified by inconsistent regulatory standards. This study aimed to assess the extent of heavy metal contamination in adaptogenic supplements on the Polish market and evaluate their compliance with international safety limits. Eleven commercially available supplements (tablets, powders, dried materials) containing *Withania somnifera*, *Rhodiola rosea*, *Panax ginseng*, and *Schisandra chinensis* were analyzed for lead (Pb), cadmium (Cd), mercury (Hg), nickel (Ni), and other elements using flame atomic absorption spectroscopy (FAAS) and mercury analysis (AMA 254). Results demonstrated widespread contamination, primarily with Pb and Ni. In processed forms (tablets and dried fruits), Pb concentrations exceeded permissible limits by up to 235%, while Ni levels were exceeded by up to 321%. *Schisandra chinensis* preparations showed the highest contamination levels. Furthermore, raw materials from India contained significantly higher Ni concentrations than those from China (*p* < 0.01). These findings reveal that a majority of the tested supplements fail to meet toxicological safety criteria, posing a significant health risk to consumers. This underscores a critical regulatory gap and highlights the urgent need for harmonized standards and stringent quality control for dietary supplements.

## 1. Introduction

The term “adaptogen” was introduced in the 1940s by the Russian toxicologist N. V. Lazarev in the context of studies on dibazole, and subsequently developed by Brekhman and Dardymov in the 1960s. At that time, three main defining criteria were established: (1) a lack of toxicity at therapeutic doses, (2) nonspecific activity that increases resistance to diverse stress factors, and (3) the ability to normalize physiological functions regardless of the direction of deviation from the norm [1,2]. The contemporary definition describes adaptogens as plant-derived substances that modulate the organism’s response to stress through effects on the neuroendocrine–immune system, restoring homeostasis and limiting the effects of oxidative stress [3]. The mechanisms of adaptogen action are pleiotropic and include, among others, effects on the hypothalamic–pituitary–adrenal axis and modulation of the expression of genes responsible for the stress response [3]. The significance and popularity of adaptogens stem from their wide spectrum of pharmacological activities, including anti-fatigue, neuroprotective, anxiolytic, nootropic, and immunomodulatory effects, as confirmed by preclinical and clinical studies [3,4,5]. Adaptogens support metabolic balance, improve mental and physical performance, and increase tolerance to environmental and emotional stress, thereby contributing to the prevention of numerous lifestyle diseases and serving as components of dietary supplements [3,6].

The Ayurvedic sector, encompassing numerous adaptogens, was valued at USD 20.6 billion in 2021, and at USD 23.3 billion in 2022 [7]. Industry research data indicate that the global value of the Ayurvedic market will increase from USD 7.27 billion in 2021 to USD 16.23 billion in 2028, with a compound annual growth rate (CAGR) of 12%. These figures confirm the growing consumer interest in Ayurvedic products [8]. The COVID-19 pandemic also contributed to increased interest not only in immunity-supporting supplements but also in adaptogenic preparations [9]. The most commonly used and best-studied adaptogens include *Panax ginseng*, *Eleutherococcus senticosus*, *Rhodiola rosea*, and *Schisandra chinensis* [6]. Thus, from a long-term perspective, adaptogens align with the growing trend of functional foods and nutraceuticals, supporting the prevention of lifestyle diseases such as obesity, diabetes, and cardiovascular disorders [6,10].

A serious threat to consumer health is the heavy metal contamination of plant raw materials [11,12,13]. Soil contamination with elements such as lead (Pb), cadmium (Cd), arsenic (As), mercury (Hg), nickel (Ni), and chromium (Cr) constitutes a global environmental problem, resulting from both natural and anthropogenic processes [14]. The toxicological relevance of these elements is well documented. Pb is a cumulative neurotoxin that can cause hypertension, disrupt endocrine and immune systems, and impair intellectual capacity in children, while also affecting renal and cardiac function in adults [15]. Cd is characterized by its high mobility in the food chain and is implicated in disorders of the cardiovascular system, kidneys, bones, and brain [15]. Both Ni and As are associated with a high cancer risk contribution, with Ni being a potent contact allergen and Cr exhibiting DNA-damaging and mutagenic potential [15,16]. Even essential elements such as Cu and Zn can exert toxic effects when consumed in excess; elevated intake of Cu may lead to liver damage, while excessive Zn can impair immune function [15]. The main sources of contamination include mineral and organic fertilizers, pesticides, and wastewater, which contribute to exceeding permissible limits of Cd and Pb in agricultural soils [14]. Industrial emissions and atmospheric deposition also represent significant sources of pollution [17]. Studies have shown that in mining regions of China, Pb and Zn concentrations were 56 and 47 times higher, respectively, than the global average, whereas in Australia, phosphate fertilizers may contain Cd ranging from 300 mg kg^−1^ up to 500 mg kg^−1^ depending on the source [18]. These contaminants also have significant toxicological implications, as their bioaccumulation increases the risk to consumer health [12,19]. Importantly, an analysis by Luo et al. of 1773 herbal samples listed in the Chinese Pharmacopoeia showed that 30.5% contained at least one metal above permissible limits. The most frequently detected elements were Pb, Cd, As, and Hg, with the proportion of samples exceeding the limits being Pb 5.75%, Cd 4.96%, As 4.17%, Hg 3.78%, and Cu 1.75% [20]. Regional studies also confirm the scale of the problem in the UAE, where 64% of the analyzed herbal samples exceeded Pb limits [21]. In the study by Rojas et al. of 26 products containing Ginkgo biloba from the Mexico City area, all analyzed products contained Pb, 54% contained As, and 81% contained Cd [22]. Comparative studies by Ćwieląg-Drabek et al. demonstrated that contamination occurred more frequently in supplements from microalgae (88.2%) than in those from terrestrial plants [23]. Moreover, even in cases of low contamination levels in individual supplements, the health risk is compounded by cumulative exposure resulting from the concurrent use of multiple herbal and dietary products [24]. These data confirm the scale of the issue, which indirectly results from the lack of global standardization, and at the same time indicate the need for more effective regulatory mechanisms and quality control in the dietary supplements industry [22].

It is important to note that dietary supplements and herbal medicinal products are regulated under different legal frameworks in the European Union (EU), which leads to significant differences in safety and quality. Supplements are classified as food and subject to Directive 2002/46/EC [25], whereas herbal medicinal products are treated as medicinal products under Directive 2001/83/EC [26]. The absence of mandatory clinical trials for supplements means that these products may be marketed without evidence of efficacy and safety, and the applicable heavy metal limits are less restrictive than those set out in EMA/HMPC herbal monographs [27]. In practice, this leads to situations where supplements may contain higher levels of Pb, Cd, or As compared with analogous preparations registered as medicines. Furthermore, the lack of unified mechanisms for monitoring the supplement market and procedures for the rapid withdrawal of contaminated batches exacerbates the problem. As a result, heavy metal contamination may only be detected at the level of academic research or clinical case reports, which underscores the importance of further toxicological studies and systematic quality monitoring [20,23].

The convergence of inconsistent regulatory frameworks and the rising popularity of adaptogenic supplements creates a significant public health concern regarding heavy metal exposure. However, the actual contamination levels in products available on specific European markets, such as Poland, remain poorly documented. Therefore, this investigation was designed to address this gap. We quantified the concentrations of lead, cadmium, arsenic, mercury, chromium, and nickel in 11 commercially available adaptogenic supplements and assessed their compliance with established pharmacopeial and international standards (EMA/HMPC, EFSA, WHO, USP). By systematically analyzing key adaptogenic species (*Panax ginseng*, *Withania somnifera*, *Rhodiola rosea*, *Eleutherococcus senticosus*, and *Schisandra chinensis*), this study provides needed data to highlight regulatory shortcomings and inform risk assessment.

## 2. Materials and Methods

### 2.1. Characteristics of the Analyzed Dietary Supplements

The study design employed a purposive sampling strategy to ensure market representativeness. Eleven dietary supplements containing adaptogenic plants, available on the Polish pharmaceutical market in 2024 (in brick-and-mortar and/or online pharmacies), were selected for the study. The selection criterion was the popularity of the preparations and their wide availability in retail distribution. The selection was not randomized but targeted to include market-leading products from different manufacturers. Care was taken to ensure sample representativeness in terms of various pharmaceutical forms (powders, capsules, tablets, dried plant raw materials) and different manufacturers, so as to account for potential technological and quality variability. As the analyzed products are processed and shelf-stable dietary supplements, seasonal variability of raw material harvest was not considered a confounding factor influencing the study design.

To ensure repeatability of the study, each sample was anonymized and assigned a unique code. All analyzed supplements were within their shelf life. Table 1 presents the sample code, plant species, pharmaceutical form, and country of raw material origin. Manufacturer data were deliberately removed to avoid commercial identification.

### 2.2. Sample Preparation

Samples were prepared according to the requirements of the applied analytical techniques. Tablets and dried raw materials were pulverized in an agate mortar, then homogenized and sieved through a 0.5 mm mesh to obtain a uniform analytical fraction. All laboratory vessels (glass and plastic) were cleaned by soaking for 24 h in a 10% solution of nitric acid (V) (HNO_3_ suprapur, Merck, Darmstadt, Germany), followed by triple rinsing with deionized water (18.2 MΩ·cm, Milli-Q system, Merck Millipore, Burlington, MA, USA) and drying at 110 °C. This procedure was critical to minimize the risk of cross-contamination, a factor known to significantly impact the accuracy of trace element analysis in complex matrices [28,29]. Only reagents of purity suitable for trace analysis were used for sample preparation (HNO_3_ 65% suprapur, Chempur, Piekary Śląskie, Poland; H_2_O_2_ 30% p.a., Chempur, Piekary Śląskie, Poland). For analyses by atomic absorption spectroscopy (AAS), 0.500 ± 0.001 g of sample was weighed in triplicate using an analytical balance (model AS 220.R2, Radwag, Radom, Poland) with an accuracy of ±0.01 mg and then transferred to Teflon digestion vessels (Nalgene, Rochester, NY, USA). For mercury determinations by the AMA 254 method, 40.0 ± 0.1 mg of micronized material was weighed using a microbalance (model MYA 5.5Y, Radwag, Radom, Poland). All operations were performed with acid-resistant tools (PTFE spatulas, Thermo Fisher Scientific, Waltham, MA, USA) and in nitrile gloves to avoid secondary contamination.

### 2.3. Scanning Electron Microscopy Analysis (SEM-EDS)

To assess surface morphology and the qualitative elemental composition of the samples, a scanning electron microscope (SEM) with an EDS attachment was used as a complementary technique. Qualitative and semi-quantitative analyses were performed using a Hitachi TM 3000 scanning electron microscope (Hitachi High-Tech Corporation, Tokyo, Japan) with an EDS attachment to determine surface morphology and identify the presence of heavy metals. Plant raw materials were mounted on the stage using conductive carbon tape (e.g., Nisshin EM Co., Ltd., Tokyo, Japan) (height < 25 mm, radius < 7 mm). Due to the lack of electrical conductivity, samples were sputter-coated with a thin layer of silver (1A, 2A, 3A, 1R, 2R, 1Z, 2Z, 1C, 2C) or platinum (3R, 3Z). The process included degassing in argon vacuum (1 × 10^−3^ mbar, 10 min), followed by sputtering for 2 min in two repetitions. Analyses were conducted at a 500× magnification, a 20 kV accelerating voltage, and a working distance of D11.1–D8.2, and the EDS detector (Quantax 70, Bruker, Berlin, Germany) recorded spectra for 120 s. Elemental distribution maps and estimated contents were obtained. Due to the possibility of result distortion caused by spectral overlap of Cd with silver and Hg with platinum, analyses of Cd and Hg content were performed on samples sputtered with platinum and/or silver, respectively.

### 2.4. Atomic Absorption Spectroscopy (AAS) Analysis

For quantitative determinations, flame atomic absorption spectroscopy (FAAS) was selected, dictated by its availability, procedural simplicity, and the ability to determine several elements simultaneously in a short time, which makes this technique a useful screening tool. Quantitative determinations of Mn, Fe, Ni, Cu, Zn, Cd, and Pb were performed using an iCE 3500 atomic absorption spectrometer (Thermo Fisher Scientific, Waltham, MA, USA) in flame mode. For wet decomposition, samples were digested in a Speedwave 4 microwave digestion system (Berghof Products + Instruments GmbH, Eningen, Germany). To each 0.500 ± 0.001 g portions, 5 mL HNO_3_ (65%, suprapur, Chempur) and 3 mL H_2_O_2_ (30%, p.a., Chempur) were added. The digestion was performed using a two-step temperature program: (1) ramping to 180 °C over 15 min, and (2) holding at 180 °C for 30 min, with the pressure maintained at 30 bar. After the program completion, the vessels were allowed to cool to room temperature. The resulting clear solutions were quantitatively transferred and filtered by gravity into 10 mL Class A volumetric flasks (Duran, DWK Life Sciences, Mainz, Germany), then brought to volume with deionized water. Fresh five-point calibration curves were prepared for each element; linearity of R^2^ ≥ 0.999 was required. Results were expressed in mg/kg d.m. Limits of detection and quantification were consistent with instrumental characteristics (IDL/IQL) and were as follows: Mn 0.0016/0.020 mg/L; Fe 0.0043/0.050 mg/L; Ni 0.0043/0.050 mg/L; Cu 0.0045/0.033 mg/L; Zn 0.0033/0.010 mg/L; Cd 0.0028/0.013 mg/L; Pb 0.0130/0.070 mg/L. Conversions from milligrams of element to kilograms of sample dry matter (mg/kg d.m.) were performed taking into account the weighed portion and the final solution volume. To verify the accuracy of the entire analytical procedure.

### 2.5. Mercury Analysis by AMA 254

Total Hg content was determined using an AMA 254 Advanced Mercury Analyzer (Altec, Prague, Czech Republic) in direct combustion mode. Micronized samples of 40 mg were dried and combusted in an oxygen stream, and Hg vapor was measured by atomic absorption at 254 nm. Results were expressed in mg/kg d.m. Instrument sensitivity was defined according to method validation, with LOD = 0.003 ng, LOQ = 0.01 ng (equivalently 0.03 and 0.1 µg/L for measurement in solution phase).

### 2.6. Method Validation and Quality Control

The analytical method was validated in terms of instrumental performance, accuracy, and precision. The instrument detection limits (IDL) and instrument quantification limits (IQL) for the iCE 3500 spectrometer were established based on the manufacturer’s specifications and are presented in Table 2.

Method accuracy was verified using the certified reference material BCR-414 (Plankton) from the Institute for Reference Materials and Measurements (Belgium). The measured concentrations were compared with the certified values, and the relative deviation was calculated. The results, summarized in Table 3, show deviations not exceeding −5.5%, confirming high analytical accuracy.

The precision of the measurements was assessed by calculating the relative standard deviation (RSD) for analytical replicates of the supplement samples. The mean RSD values for all analyzed elements were below 2.5%, indicating measurement repeatability (Table 4).

The linearity of the method for each element was confirmed for all calibration curves, which are provided in the Supplementary Material (Figures S1–S7).

### 2.7. Comparison of Obtained Results with Reference Values and Statistical Analysis

The obtained concentrations of heavy metals in the analyzed dietary supplements were compared with reference values specified in applicable normative documents. For elements such as Pb, Cd, Hg, and Ni, the reference points were the limits of heavy metals content in plant raw materials and medicinal preparations indicated in the European Pharmacopoeia [30], as well as the permissible values published in the WHO guidelines on the quality of plant raw materials [31]. In addition, risk assessment criteria developed by the European Food Safety Authority (EFSA) were taken into account, including the Tolerable Weekly Intake (TWI) and the Benchmark Dose Lower Confidence Limit (BMDL) for Pb and Cd [32,33].

In the case of biologically essential trace elements (Mn, Fe, Cu, Zn), comparisons were made with reference values established by the EFSA Scientific Committee for Tolerable Upper Intake Levels (ULs) for adults [34], which allowed assessment of whether the determined content could lead to the permissible level of daily intake being exceeded.

All quantitative determinations were carried out in triplicate, after which arithmetic means with standard deviations were calculated and presented in tables as values representative of a given preparation. The obtained data were used for direct comparison with toxicological and pharmacopeial standards. Results obtained by SEM/EDS, due to the surface nature of the analysis and limited quantitative repeatability, were treated as qualitative–semi-quantitative—indicating only the presence and distribution of elements in the analyzed surface layer of the sample, without the possibility of extrapolation to its entire mass.

Statistical analysis was performed using Statistica 13.3 software (TIBCO Software Inc., Palo Alto, CA, USA). To assess the effect of the geographical origin of the raw material (India vs. China) on metal concentrations, an independent Student’s *t*-test was applied. To examine differences in element concentrations between different pharmaceutical forms (capsule, tablet, root, fruit), one-way analysis of variance (ANOVA) was used, and the LSD post hoc test was applied for detailed intergroup comparisons. Additionally, to investigate relationships between the concentrations of individual metals, the Pearson correlation coefficient (r) was calculated. In all tests, a significance level of *p* < 0.05 was adopted.

## 3. Results

### 3.1. Analysis of Morphology and Elemental Composition of Dietary Supplements (SEM/EDS)

**Figure 1:**
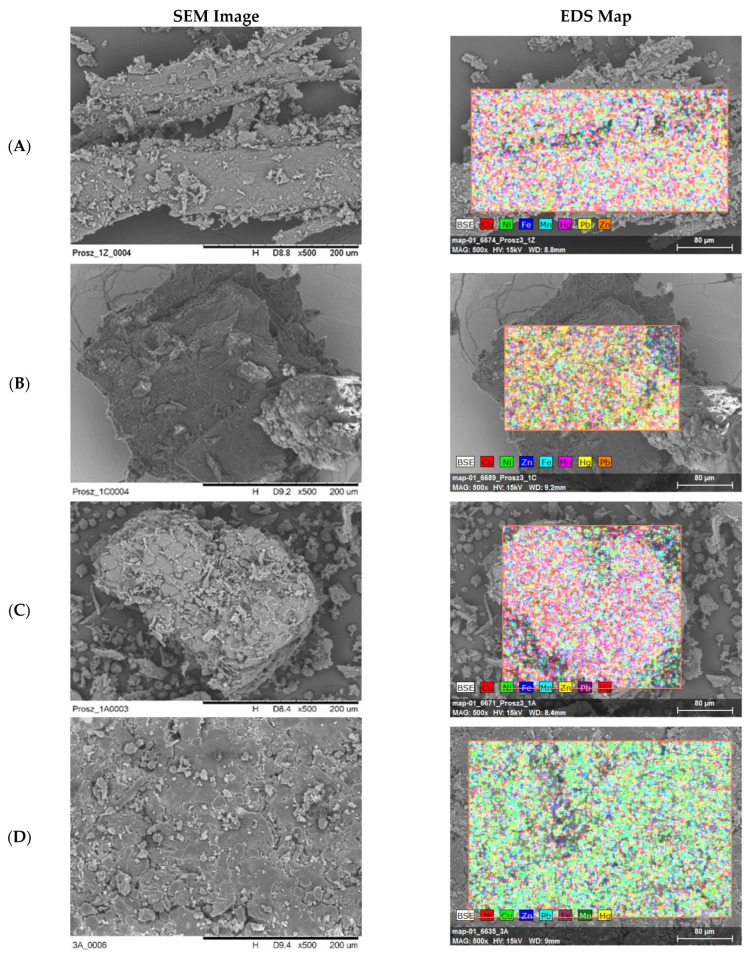
The image SEM of surface morphology of the analyzed dietary supplements depending on the pharmaceutical form. (**A**) Natural, fibrous structure of dried ginseng root (sample 1Z); (**B**) Wrinkled surface of dried *Schisandra chinensis* fruit (sample 1C); (**C**) Irregular particles of powdered *Withania somnifera* root (sample 1A), formed as a result of grinding; (**D**) Strongly compressed and agglomerated structure of an ashwagandha tablet (sample 3A), illustrating the effect of technological processing.

Preliminary morphological and qualitative analysis performed using scanning electron microscopy with energy-dispersive X-ray microanalysis (SEM/EDS) enabled the assessment of the texture of the tested samples and the detection of the presence of heavy metals. All examined dietary supplements-regardless of the form (powders, capsules, tablets, dried fruits and roots)—exhibited a porous, irregular, and multilayered structure, often forming compact agglomerates, particularly in the case of powders (Figure 1). Such surface configuration favored potential adsorption of contaminants and indicated possible variation in the distribution of metals within a single sample.

EDS analysis unequivocally confirmed the presence of heavy metals in all dietary supplements, with Pb and Hg being the dominant elements, and with additional presence of Ni, Cu, Mn, Zn, and Fe. In supplements containing ashwagandha, a predominance of Pb and a significant share of Hg were found, alongside trace amounts of Cu and Ni. In the case of *Rhodiola rosea*, Pb predominated (1R, 3R), whereas in sample 2R Hg had the largest share. In this group of samples, Cu, Zn, Ni, and Fe were additionally detected. Korean ginseng supplements showed the greatest shares of Pb and Hg, and in each sample small amounts of Ni and Mn were also recorded. In *Schisandra chinensis* preparations, the dominant elements were Pb and Hg, alongside trace amounts of Cu, Fe, Mn, and Ni. It should be emphasized that SEM/EDS analysis provided only qualitative and semi-quantitative information referring to the examined surface, and not to the entire sample.

### 3.2. Quantitative Analysis of Heavy Metals Content (AAS and AMA)

Precise quantitative determinations of the discussed elements were performed using atomic absorption spectroscopy (AAS) and the AMA 254 mercury analyser. The obtained averaged values are presented in Table 5 and were compared with the permissible limits contained in the European Pharmacopoeia, WHO guidelines, and EFSA, taking into account Maximum Permissible Levels (MPL), Tolerable Upper Intake Levels (UL), and specific limits for plant raw materials. Comparative standards are compiled in Table 6.

In none of the examined supplements were exceedances of Cd and Hg observed, nor of Cu, Zn, and Fe with respect to the recommended daily doses. The permissible standards were most frequently exceeded for Pb and Ni, and in single cases also for Mn (data highlighted in bold in Table 5). To fully assess the safety of the tested dietary supplements, the results of quantitative determinations of heavy metals content were compared with the applicable legal and toxicological standards. Table 7 presents a comparison of Pb and Ni concentrations in our samples with reference values, indicating unambiguously which preparations exceeded the permissible limits. The problem of exceeding standards concerned primarily two elements, i.e., Pb and Ni (as indicated in Table 5). The percentage level of exceedance relative to the permissible values was also included, which allows better illustration of the scale of the health risk associated with the use of individual products. The demonstrated excessive amounts of Pb and Ni were observed in the majority of samples, and the degree of exceedances in many cases significantly exceeded the threshold values defined in the European Pharmacopoeia and the EFSA and WHO guidelines.

### 3.3. Impact of Pharmaceutical Form and Raw Material Origin on the Profile of Metals Contamination

The analysis of results showed that the supplement form affected the degree of contamination by metals. Excessive amounts of Pb occurred more frequently in tablets, which may result from additional technological processes and excipients used. Ni contamination was independent of the form, which suggests that the main source was contaminated plant material. The least contaminated were preparations in the form of dried root, which confirms the hypothesis of a lesser impact of processing. To confirm the above observations, a one-way analysis of variance (ANOVA) was performed to assess the effect of product form on metal concentrations in the tested supplements. The analysis results showed a statistically significant effect of product form on the elemental profile (*p* < 0.001, F = 18.030). Detailed post hoc analysis (LSD test) revealed that processed products (tablets and capsules) were characterized by statistically significantly higher Pb concentrations compared with unprocessed raw materials (roots and fruits), which indicates technological processing as a potential source of contamination. In the case of Mn and Hg, the highest concentrations were recorded in roots/fruits and fruits, respectively, which suggests the impact of anthropogenic processes on the quality of plant material. Importantly, for Ni, Cu, Zn, and Cd, no statistically significant differences were found between the individual forms, which suggests that their presence is related to primary contamination of the plant raw material rather than the processing stage.

An important factor differentiating the level of contamination was also the origin of the raw material. To assess the effect of the country of origin on the level of contamination, a Student’s *t*-test was performed, comparing metal concentrations in samples originating from India and China. The analysis showed a highly statistically significant difference in the elemental profile between the two groups (T^2^ = 384.22; *p* < 0.001), which indicates the importance of geographical origin for the metal content in the tested supplements. Analysis of individual elements revealed that supplements whose raw material originated from India were characterized by statistically significantly higher concentrations of Cu, Zn, and Ni (*p* < 0.01 for all). In particular, the mean concentration of nickel (Ni), an element for which numerous exceedances were recorded, was more than twice as high in Indian samples (1.57 mg/kg) as in Chinese samples (0.79 mg/kg). For Mn, higher concentrations were observed in samples from China (32.56 mg/kg) compared with samples from India (13.73 mg/kg), with this difference at the threshold of statistical significance (*p* = 0.056). Importantly, for metals of greatest toxicological significance, such as Pb (*p* = 0.75), Cd (*p* = 0.30), and Hg (*p* = 0.30), no statistically significant differences in concentrations were found between the two geographical groups. This suggests that the problem of contamination with these elements is global in nature and is not limited to one region in the studied group of products.

To identify patterns of covariation of heavy metals concentrations in the tested dietary supplements and to infer their common potential sources of contamination, Pearson correlation analysis was performed. The strongest positive relationship was observed between Cu and Zn (r = 0.87), which unequivocally indicates their common source of origin. Similarly strong correlations were observed within the Cu–Zn–Ni group, i.e., between Zn and Ni (r = 0.66) and Cu and Ni (r = 0.63). The high coherence of these three elements suggests that they may originate from similar anthropogenic sources, such as industrial pollution, metallurgical processes, or the use of mineral fertilizers. Additionally, moderately strong correlations were demonstrated between Fe and Pb (r = 0.61) and Mn and Hg (r = 0.59). In the first case, the relationship may indicate common atmospheric deposition or soil contamination, whereas in the second it may indicate similar soil properties determining the bioavailability of both elements. It is worth emphasizing that Cd did not show significant correlations with any other element, which suggests a different mechanism for its introduction into the tested material.

## 4. Discussion

The results of this study provide alarming evidence of widespread and significant heavy metals contamination of dietary supplements containing adaptogens available on the Polish market. The identified exceedances of standards for Pb and nickel in most of the tested preparations constitute a significant public health hazard and reveal critical gaps in quality control systems for these products. Pb and nickel constitute the main threat in the examined group of supplements. The vast majority of analyzed products did not meet safety criteria regarding the content of these two metals. These findings align with the global problem of heavy metal contamination in herbal raw materials indicated, among others, by Luo et al. [20]. Particularly concerning are the confirmed high concentrations of Pb, an element with documented neurotoxicity, cardiotoxicity, and adverse effects on development, even at low levels of exposure [42,43]. The exceedances of standards found in preparations 3A (tablets containing ashwagandha), 3Z (tablets containing ginseng), 1C (dried fruits of *Schisandra chinensis*), and 2C (tablets containing *Schisandra chinensis*), ranging from 70% to as much as 235%, indicate a serious risk to consumers, especially with long-term use. This alarming scale is corroborated by regional studies, such as the analysis of herbal samples in the UAE, where 64% were found to exceed Pb limits [21], and studies in Mexico City, where all tested Ginkgo biloba products contained detectable Pb [22].

Equally important is the problem of excessive nickel content, particularly evident in all ashwagandha samples (1A, 2A, 3A) and in preparations of *Schisandra chinensis* (1C, 2C). Nickel is a strong contact allergen, and its excessive intake is associated with metabolic disorders and potential carcinogenic effects [44]. The clinical relevance of this exposure is highlighted by human biomonitoring data, which show that the “ingestion of dietary supplements” is a significant predictor of increased nickel excretion in urine, confirming that Ni from supplements contributes to the total body burden [45]. Notably, high nickel concentrations have been previously reported in supplements on the Polish market, with levels in ginseng-based products reaching up to 4.10 mg/kg, which corroborates our findings of systemic contamination within this specific product category [46].

Beyond the direct health risks, our findings translate into specific, practical implications for quality control across the supply chain. The results critically distinguish between two primary contamination pathways: the quality of the raw material at its source and contaminants introduced during industrial processing.

First, the consistently elevated Ni concentrations in all *Withania somnifera* samples from India indicate a systemic environmental contamination issue at the cultivation stage, likely related to contaminated soils or irrigation waters [47]. Such contamination may stem from agricultural inputs like phosphate fertilizers and pesticides, or from industrial effluents [48]. This finding implies that raw materials sourced from certain geographical regions should be classified as high-risk and be subject to mandatory batch testing. Furthermore, it highlights the necessity of implementing and enforcing Good Agricultural and Collection Practices (GACP) in sourcing regions, which include monitoring of soil, water, and control over agricultural inputs to ensure the safety of raw materials at the source [49,50].

Second, the statistically significant higher concentrations of Pb in processed products (tablets) confirm that manufacturing is a key secondary contamination source. This may be attributed to either the use of contaminated excipients or the leaching of metals from production line components [51]. The strong positive correlation observed between iron (Fe) and Pb (r = 0.61) may also suggest a common source, such as atmospheric deposition from industrial emissions in proximity to manufacturing or cultivation sites. These observations underscore the absolute necessity of implementing and enforcing stringent GMP standards in the dietary supplement industry. Reliance on raw material testing alone is insufficient; safety assurance requires validating the purity of all excipients and monitoring the entire technological process to prevent secondary product contamination [50].

The fact that nickel content in root-based raw materials (1A, 2A, 3A, 1Z) and fruit-based materials (1C, 2C) exceeded standards by 35% to 320% indicates a problem with the quality of plant raw materials used in the production of dietary supplements. The potential source of contamination may therefore be both contaminated plant material and processing procedures. It was shown that the mean nickel concentration in samples from India (1.57 mg/kg) was more than twice as high (*p* < 0.01) as the concentrations in samples from China (0.79 mg/kg). This is consistent with reports of elevated nickel levels in medicinal plants from Asian regions, often linked to contaminated soil and industrial activity [12,14]. This geographical discrepancy is not an isolated finding. Research on supplements available in the UAE market specifically identified that products made in India pose a “high risk of being contaminated with lead” and other metals. This may be linked to certain practices in traditional Indian medicine, where it is sometimes believed that heavy metals are required for proper therapeutic functioning, leading to their potential deliberate addition [52].

The detection of excessive nickel in all ashwagandha samples originating from India, regardless of the form of the preparation, indicates an environmental contamination problem (soil, water, air) at the cultivation site. Thus, sources of metals such as fertilizers, pesticides, or industrial emissions may affect the level of soil contamination, contributing to the presence of heavy metals in plant raw materials [14]. Very strong positive correlations were also found within the Cu–Zn–Ni group (r = 0.63–0.87), which unequivocally indicates their common, probably industrial or agricultural, source of origin. The moderately strong association between Fe and Pb (r = 0.61) may in turn suggest atmospheric deposition as a common route of contamination.

Conversely, the high concentrations of Pb, predominant in preparations in tablet form (3A, 2R, 3Z), may result from contaminants introduced at the production stage, where control in the case of dietary supplements is less stringent compared to medicines (OTC). This is in line with previous findings where manufacturing processes, particularly the use of contaminated excipients (fillers, binders) or cross-contamination from production lines, were identified as sources of heavy metal contamination in finished products [23,53]. This hypothesis is supported by evidence from process-monitoring studies; for instance, in the production of yam powder, Ni content was observed to increase specifically after the grinding stage, indicating contamination from processing equipment [54]. Furthermore, analyses of various medicinal agents have led to the conclusion that while raw materials are a primary source, the manufacturing processes themselves are a major contributor to the presence of toxic trace metal contaminants [55]. It was shown that processed products, i.e., tablets and capsules, were characterized by statistically significantly higher concentrations of Pb (*p* < 0.001) compared with unprocessed raw materials (roots and fruits). The absence of stringent Good Manufacturing Practice (GMP) requirements for dietary supplements may also lead to the use of contaminated excipients (fillers, binders) or cross-contamination from production lines [56]. Our study clearly demonstrated that *Schisandra chinensis* was the most contaminated raw material in the analyzed group. In both analyzed samples (1C and 2C), multiple exceedances of standards for Pb and nickel were found, which makes them products with the highest toxicological risk. This is particularly alarming in the context of the growing popularity of this adaptogen, valued for its hepatoprotective properties and support of the nervous system [53]. These results call into question the safety of using supplements based on this raw material and underscore the need for its special monitoring. It is worth noting that, paradoxically, consumers turn to adaptogens to improve health and increase resistance to stress [4,57], thereby potentially exposing themselves unknowingly to the toxic effects of heavy metals.

The presented results constitute a strong argument for the thesis that classifying dietary supplements as food rather than medicinal products leads to inadequate consumer protection. The legal framework set out in Directive 2002/46/EC for dietary supplements is significantly less restrictive than that for herbal medicines (Directive 2001/83/EC) [58]. This leads to a situation where products of unconfirmed quality, potentially hazardous to health, may enter the market. As indicated, contamination limits for supplements are often less stringent than those present in EMA/HMPC herbal monographs, which in practice legalizes the presence of higher concentrations of toxic metals [20]. This problem reveals an urgent need to harmonize requirements for supplements and herbal medicines in favor of implementing more rigorous limits and introducing an obligation for systematic quality monitoring in the dietary supplements industry [22]. Importantly, the contamination found in the tested products is in stark contradiction to the fundamental definition of an adaptogen, the first criterion of which is “lack of toxicity at therapeutic doses” [1].

It is worth noting that the authors are aware of certain limitations of this study. The investigation was based on a purposive selection of eleven popular products from the Polish market, providing a market snapshot rather than a comprehensive statistical survey. As samples were drawn from single production batches, the results do not account for potential batch-to-batch variability. Methodologically, the use of FAAS for Cd and Pb quantification, while effective for detecting high-level contamination, possesses a higher limit of detection than reference methods such as ICP-MS. This implies that the presence of these metals at lower, yet still potentially toxic, concentrations may have been missed, suggesting the overall health risk could be underestimated. Finally, this study quantified total metal content; a full toxicological risk assessment would necessitate chemical speciation and bioavailability analyses to determine the precise toxic potential. Despite these constraints, which define the boundaries for data interpretation, the findings provide evidence of a significant public health problem and constitute a scientific basis for urgent regulatory attention and broader monitoring studies.

## 5. Conclusions

This study provides direct evidence that a majority of tested adaptogenic supplements on the Polish market are contaminated with lead and nickel to levels that pose a significant health risk. The research results demonstrate that contamination pathways are twofold. First, the highest Pb concentrations were found in tablets, indicating that industrial processing and the use of excipients are a critical source of contamination. Second, consistent exceedances of Ni standards in all ashwagandha samples originating from India point to a systemic environmental contamination problem at the cultivation stage. *Schisandra chinensis* was identified as the most contaminated raw material, with preparations exceeding Pb and Ni limits by more than three times. These findings lead to an unequivocal conclusion: the current regulatory framework, which classifies supplements as food, is inadequate and facilitates the entry of hazardous products onto the market. Consequently, a safer alternative for consumers seeking adaptogenic therapy is to choose products registered as herbal medicines, which are subject to more stringent, pharmacopeial quality standards.

## Figures and Tables

**Table 1:** Characteristics of the analyzed dietary supplement samples.

Sample Code	Adaptogenic Plant Species	Pharmaceutical Form	Country of Raw Material Origin
1A	*Withania somnifera* (Ashwagandha)	Powder	India
2A	*Withania somnifera* (Ashwagandha)	Capsule	India
3A	*Withania somnifera* (Ashwagandha)	Tablet	India
1R	*Rhodiola rosea* (Rhodiola)	Dried root	China
2R	*Rhodiola rosea* (Rhodiola)	Tablet	China
3R	*Rhodiola rosea* (Rhodiola)	Capsule	China
1Z	*Panax ginseng* (Korean ginseng)	Dried root	China
2Z	*Panax ginseng* (Korean ginseng)	Dried root	China
3Z	*Panax ginseng* (Korean ginseng)	Tablet	China
1C	*Schisandra chinensis* (Schisandra)	Dried fruit	China
2C	*Schisandra chinensis* (Schisandra)	Tablet	China

**Table 2:** Detection limits (IDL) and quantification limits (IQL) characterizing the iCE 3500 spectrometer [mg/L].

Metal	IDL	IQL
Mn	0.0016	0.020
Fe	0.0043	0.050
Ni	0.0043	0.050
Cu	0.0045	0.033
Zn	0.0033	0.010
Cd	0.0028	0.013
Pb	0.0130	0.070

**Table 3:** Comparison of measured and certified analyte concentrations in BCR-414 plankton.

Metal	BCR-414 Plankton Concentration ± Uncertainty [mg/kg d.m.]	AAS Mean ± SD * [mg/kg d.m.]	Dev. [%] **
Mn	299 ± 12	284 ± 13	−5.0
Fe	1.85 ± 0.19	1.79 ± 0.20	−3.2
Ni	18.8 ± 0.8	18.2 ± 0.9	−3.2
Cu	29.5 ± 1.3	28.4 ± 1.6	−3.7
Zn	112 ± 3	107 ± 3	−4.5
Cd	0.383 ± 0.014	0.371 ± 0.018	−3.1
Pb	3.97 ± 0.19	3.75 ± 0.21	−5.5

*—standard deviation. **—relative difference between measured and certified concentration (100%). d.m.—dry matter.

**Table 4:** Mean RSD values [%] for analytical replicates of supplement samples.

Parameter	Mn	Fe	Ni	Cu	Zn	Cd	Pb
RSD [%]	0.17	0.32	1.12	0.48	0.18	2.14	1.54

**Table 5:** Mean concentrations of metals in the analyzed dietary supplements [mg/kg d.m.].

Sample Code	Cu	Mn	Zn	Fe	Ni	Pb	Cd	Hg
1A	4.876 ± 1.911	18.534 ± 2.573	31.192 ± 4.584	252.202 ± 65.895	**1.218 ± 0.173 ^a^**	<1.4 ^c^	0.444 ± 0.111	0.00125 ± 0.00006
2A	2.604 ± 1.021	7.746 ± 1.075	13.842 ± 2.034	53.284 ± 13.922	**1.320 ± 0.243 ^a^**	2.284 ± 0.287	0.414 ± 0.072	0.00141 ± 0.00034
3A	10.936 ± 0.429	14.906 ± 0.207	22.658 ± 0.333	21.278 ± 0.556	**2.166 ± 0.198 ^a^**	**5.146 ± 0.647 ^b^**	0.460 ± 0.080	0.00368 ± 0.00020
1R	1.618 ± 0.063	40.624 ± 0.564	8.544 ± 0.126	34.470 ± 0.901	<1 ^c^	<1.4 ^c^	0.644 ± 0.113	0.00343 ± 0.00141
2R	0.672 ± 0.026	6.420 ± 0.089	2.388 ± 0.035	28.792 ± 0.752	<1 ^c^	**3.656 ± 0.460 ^b^**	0.446 ± 0.078	0.00104 ± 0.00005
3R	2.270 ± 0.089	9.314 ± 0.129	4.888 ± 0.072	44.486 ± 1.162	**1.518 ± 0.139 ^a^**	<1.4 ^c^	0.452 ± 0.079	0.00161 ± 0.00026
1Z	2.374 ± 0.093	**68.164 ± 0.946 ^d^**	7.542 ± 0.111	28.138 ± 0.818	**1.304 ± 0.119 ^a^**	2.326 ± 0.292	0.500 ± 0.087	0.00283 ± 0.00046
2Z	2.308 ± 0.905	16.334 ± 2.267	5.758 ± 0.846	35.890 ± 9.377	<1 ^c^	1.692 ± 0.213	0.444 ± 0.078	0.00221 ± 0.00040
3Z	0.788 ± 0.031	0.960 ± 0.133	4.140 ± 0.608	36.980 ± 9.662	<1 ^c^	**7.836 ± 0.985 ^b^**	0.420 ± 0.073	0.00272 ± 0.00029
1C	4.678 ± 1.833	76.832 ± 10.665	12.746 ± 1.873	161.544 ± 42.208	**1.648 ± 0.151 ^a^**	**10.048 ± 1.263 ^b^**	0.454 ± 0.008	0.00485 ± 0.00012
2C	1.026 ± 0.040	41.832 ± 5.806	7.534 ± 1.107	207.888 ± 5.432	**1.682 ± 0.154 ^a^**	**8.192 ± 1.030 ^b^**	0.486 ± 0.008	0.00235 ± 0.00024

Footnotes: ^a^ Value exceeds the permissible limit for nickel (Ni); ^b^ Value exceeds the Maximum Permissible Level (MPL) for lead (Pb); ^c^ Value below the limit of quantification (LOQ); ^d^ Manganese concentration which, after conversion to daily intake, exceeded the maximum single intake (MSL).

**Table 6:** Regulatory limits and reference values for metal content in dietary supplements.

Element	MPL [mg/kg d.m.] [35,36,37]	MSL [mg/day] [38]	RDI in Herbal Medicines [mg/day] [39]	UL [mg/day] [40]	ML in Plant Material [mg/kg d.m.] [39,41]
Pb	≤3.0	–	0.25	–	–
Cd	≤1.0	–	0.07	–	–
Hg	≤0.1	–	–	–	–
Mn	–	2	2–5	–	–
Zn	–	10–15	15	25	–
Fe	–	14–20	–	50–60 LOAEL	–
Ni	–	–	–	–	0.4 (fruits)/0.9 (roots and tubers)
Cu	–	1–2	1.5–3	5	–

Legend: MPL—Maximum Permissible Level; MSL—Maximum Single Intake (daily dose); RDI—Recommended Daily Intake in herbal medicines; UL—Tolerable Upper Intake Level (adults); ML—Maximum Level permissible in plant raw material; d.m.—dry matter.

**Table 7:** Safety assessment of dietary supplements—comparison of toxic element concentrations with regulatory limits.

Sample Code	Element	Measured Concentration [mg/kg d.m.]	Permissible Limit [mg/kg d.m.]	Status	Exceedance [%]
1A	Ni	1.218 ± 0.173	0.9	Exceeded	35%
2A	Ni	1.320 ± 0.243	0.9	Exceeded	47%
3A	Ni	2.166 ± 0.198	0.9	Exceeded	141%
3A	Pb	5.146 ± 0.647	3.0	Exceeded	72%
2R	Pb	3.656 ± 0.460	3.0	Exceeded	22%
3R	Ni	1.518 ± 0.139	0.9	Exceeded	69%
1Z	Ni	1.304 ± 0.119	0.9	Exceeded	45%
3Z	Pb	7.836 ± 0.985	3.0	Exceeded	161%
1C	Ni	1.648 ± 0.151	0.4	Exceeded	312%
1C	Pb	10.048 ± 1.263	3.0	Exceeded	235%
2C	Ni	1.682 ± 0.154	0.4	Exceeded	321%
2C	Pb	8.192 ± 1.030	3.0	Exceeded	173%

Note: Permissible limits were adopted based on the standards presented in Table 6. For Pb, the Maximum Permissible Level (MPL) of 3.0 mg/kg, as specified in the European Pharmacopoeia for herbal drugs [36,37,38] was applied. For Ni, the Maximum Level (ML) in plant raw material was used, corresponding to 0.9 mg/kg for root/tuber-based products and 0.4 mg/kg for fruit-based products, based on international guidelines [40,41]. Legend: d.m.—dry matter.

## Data Availability

All data supporting the reported results are contained within the manuscript. Additional information may be provided upon reasonable request to the corresponding author.

## Supplementary Materials

The following supporting information can be downloaded at: https://www.mdpi.com/article/10.3390/biology14111479/s1, Figure S1. The calibration curve used for the quantification of lead (Pb); Figure S2. The calibration curve used for the quantification of cadmium (Cd); Figure S3. The calibration curve used for the quantification of manganese (Mn); Figure S4. The calibration curve used for the quantification of copper (Cu); Figure S5. The calibration curve used for the quantitative determination of iron (Fe); Figure S6. The calibration curve used for the quantitative determination of nickel (Ni); Figure S7. The calibration curve used for the quantitative determination of zinc (Zn).
