# Supplementary material for: Heavy Metal Contamination in Adaptogenic Herbal Dietary Supplements: Experimental, Assessment and Regulatory Safety Perspectives
Source: Biology (Basel). 2025 Oct 23;14(11):1479. doi: 10.3390/biology14111479 (PMC12650394; doi:10.3390/biology14111479)
Supplement: Supplementary file 1 [file biology-14-01479-s001.zip › biology-3922821-supplementary.pdf]

Supplementary Material

Heavy Metal Contamination in Adaptogenic Herbal Dietary Supplements: Experimental, Assessment and Regulatory Safety Perspectives

Agata Jasińska-Balwierz [1*], Patrycja Krypel [2], Paweł Świsłowski [3], Małgorzata Rajfur [3], Radosław Balwierz [2*], and Wioletta Ochędzan-Siodłak [2,]

[1] Department of Pharmacology, Academy of Silesia, 40-555 Katowice, Poland
[2] Institute of Chemistry, University of Opole, Oleska 48 St. Opole, Poland
[3] Institute of Biology, University of Opole, Kominka 6,6a St. Opole, Poland
* Correspondence: Agata Jasińska -Balwierz agata.jasinska.balwierz@gmail.com or Radosław Balwierz radoslaw.balwierz@uni.opole.pl;

Table of Contents

Fig. S1. The calibration curve used for the quantification of lead (Pb). 2
Fig. S2. The calibration curve used for the quantification of cadmium (Cd). 3
Fig. S3. The calibration curve used for the quantification of manganese (Mn)......................... 4
Fig. S4. The calibration curve used for the quantification of copper (Cu). 5
Fig. S5. The calibration curve used for the quantitative determination of iron (Fe). 6
Fig. S6. The calibration curve used for the quantitative determination of nickel (Ni). 7
Fig. S7. The calibration curve used for the quantitative determination of zinc (Zn)................. 8

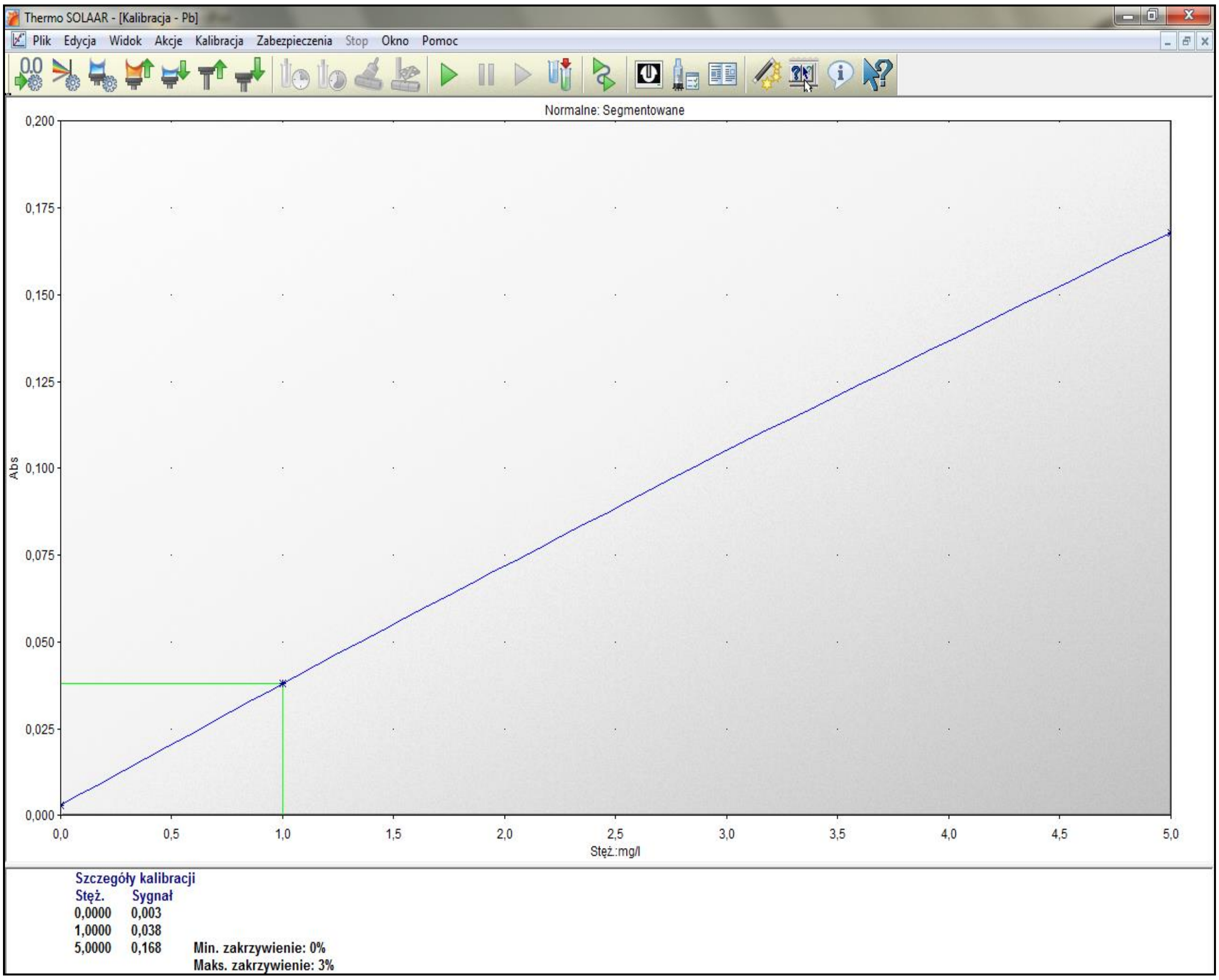

Fig. S1. The calibration curve used for the quantification of lead (Pb). The curve was generated using standards at concentrations of 0.0, 1.0, and 5.0 mg/L. The software's internal validation confirmed that the curve's deviation from linearity ("zakrzywienie") was 0%, which is well within the acceptable limit of 3%. Legend: Kalibracja (Calibration), Abs (Absorbance), Stęż. (Concentration), Szczegóły kalibracji (Calibration Details), Sygnał (Signal / Absorbance), Zakrzywienie (Curvature / Deviation from linearity).

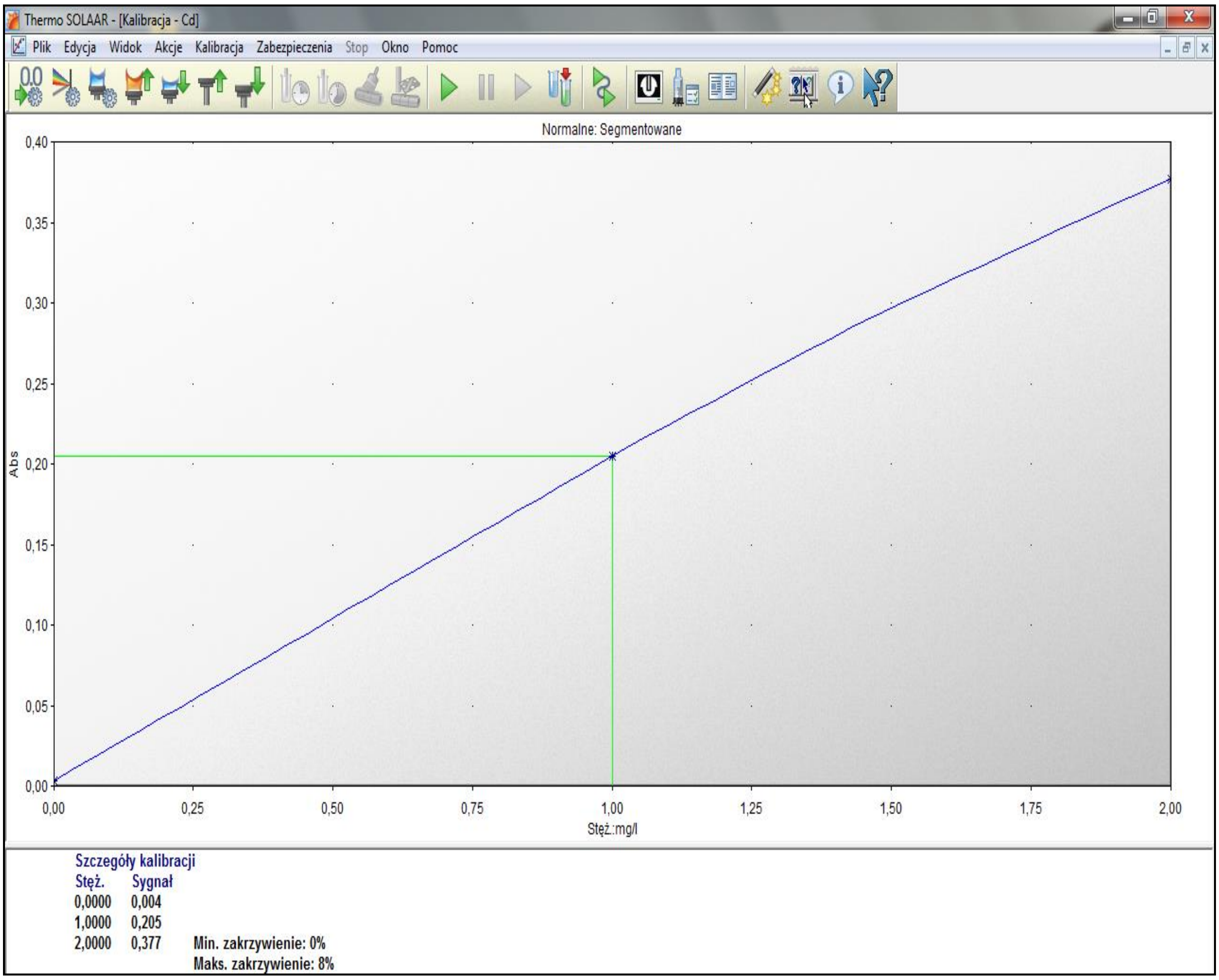

Fig. S2. The calibration curve used for the quantification of cadmium (Cd). The curve was plotted using standards at concentrations of 0.0, 1.0, and 2.0 mg/L. The software's internal validation indicated that the curve's deviation from linearity ("zakrzywienie") was 0%, which is well within the acceptable instrument limit of 8%. Legend: Kalibracja (Calibration), Abs (Absorbance), Stęż. (Concentration), Szczegóły kalibracji (Calibration Details), Sygnał (Signal / Absorbance), Zakrzywienie (Curvature / Deviation from linearity).

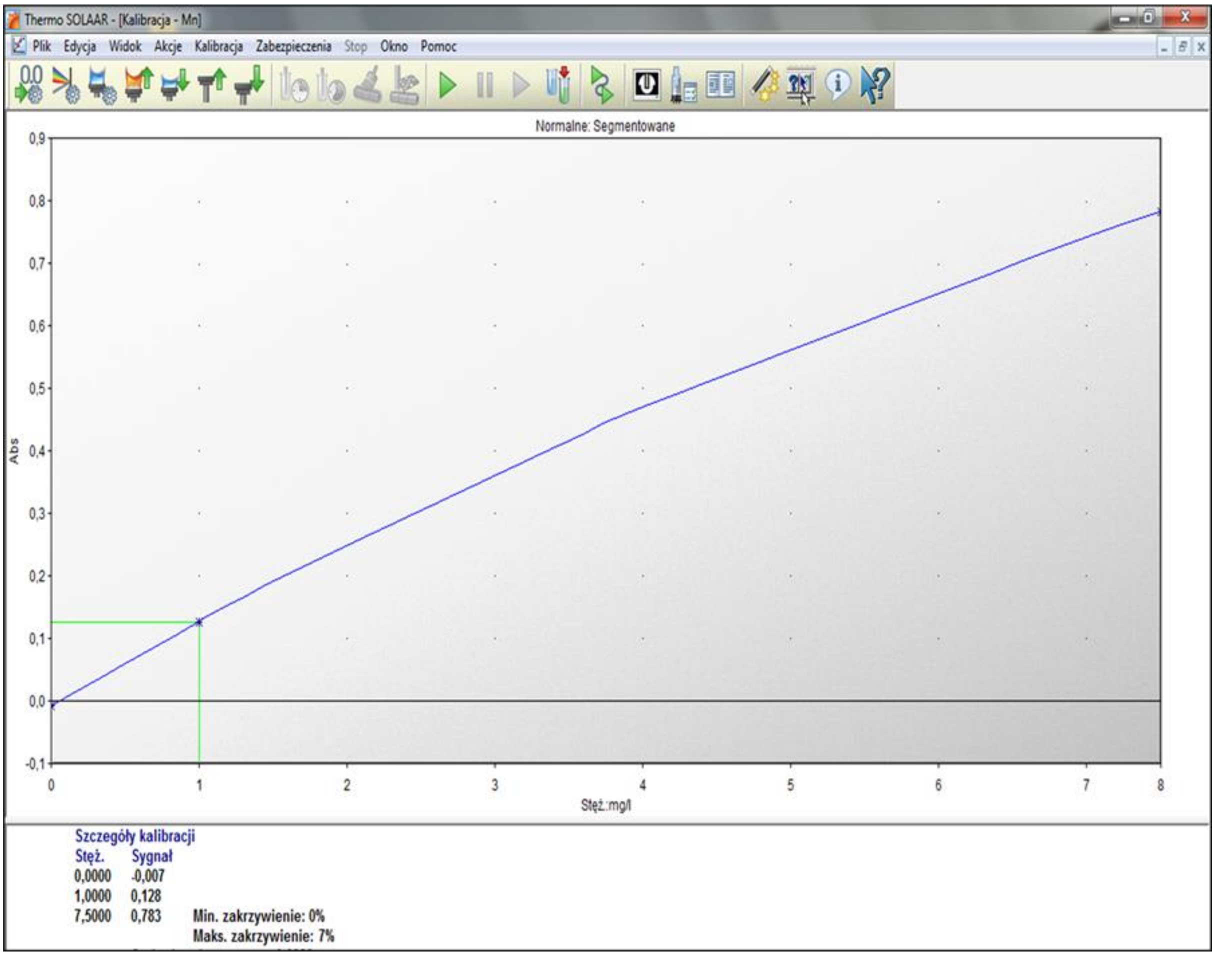

Fig. S3. The calibration curve used for the quantification of manganese (Mn). The curve was established using standards at concentrations of 0.0, 1.0, and 7.5 mg/L. The instrument software's internal validation confirmed that the curve's deviation from linearity ("zakrzywienie") was 0%, which is well within the acceptable limit of 7%. Legend: Kalibracja (Calibration), Abs (Absorbance), Stęż. (Concentration), Szczegóły kalibracji (Calibration Details), Sygnał (Signal / Absorbance), Zakrzywienie (Curvature / Deviation from linearity).

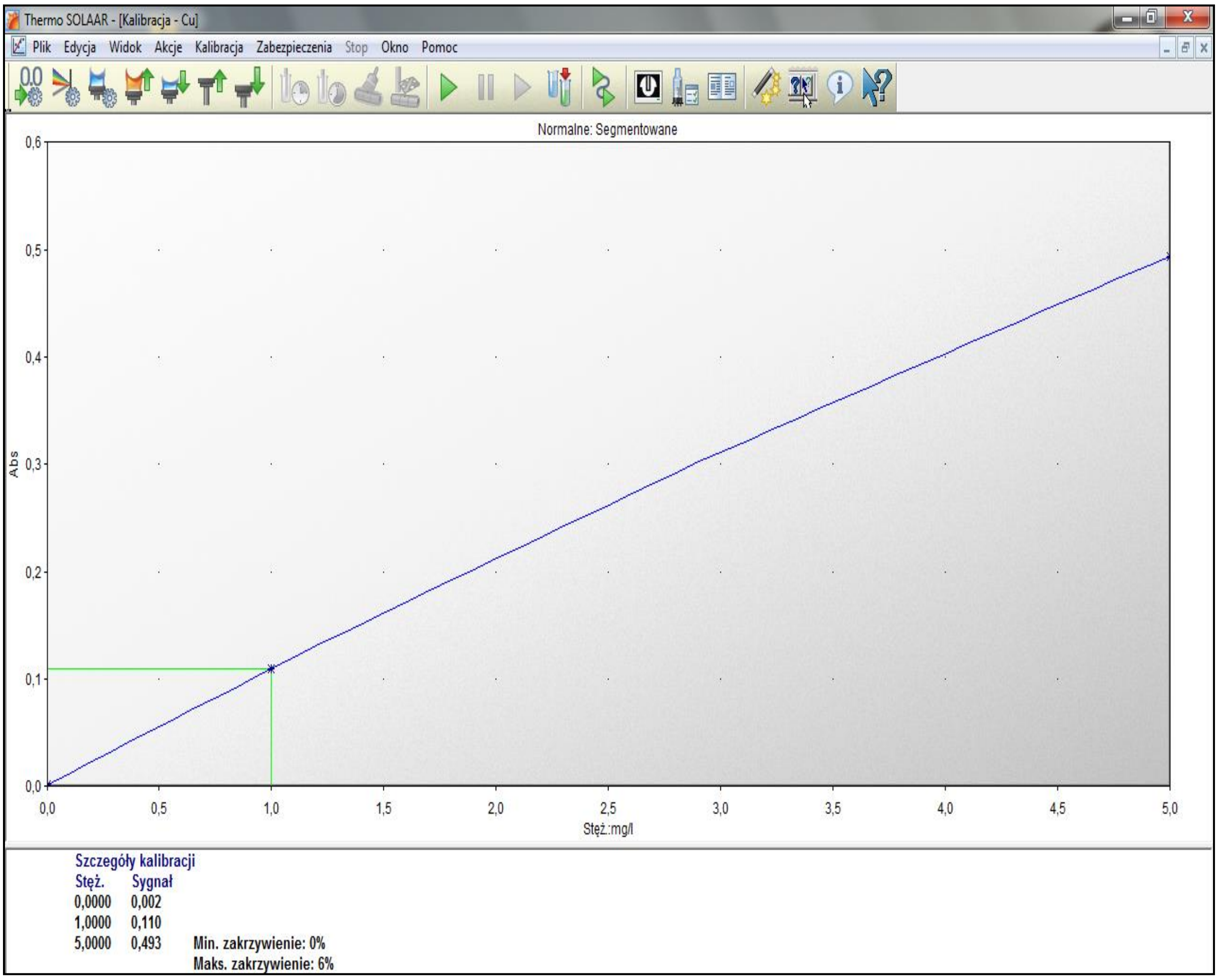

Fig. S4. The calibration curve used for the quantification of copper (Cu). The curve was generated using standards at concentrations of 0.0, 1.0, and 5.0 mg/L. The instrument software's internal validation confirmed that the curve's deviation from linearity ("zakrzywienie") was 0%, which is well within the acceptable limit of 6%. Legend: Kalibracja (Calibration), Abs (Absorbance), Stęż. (Concentration), Szczegóły kalibracji (Calibration Details), Sygnał (Signal / Absorbance), Zakrzywienie (Curvature / Deviation from linearity).

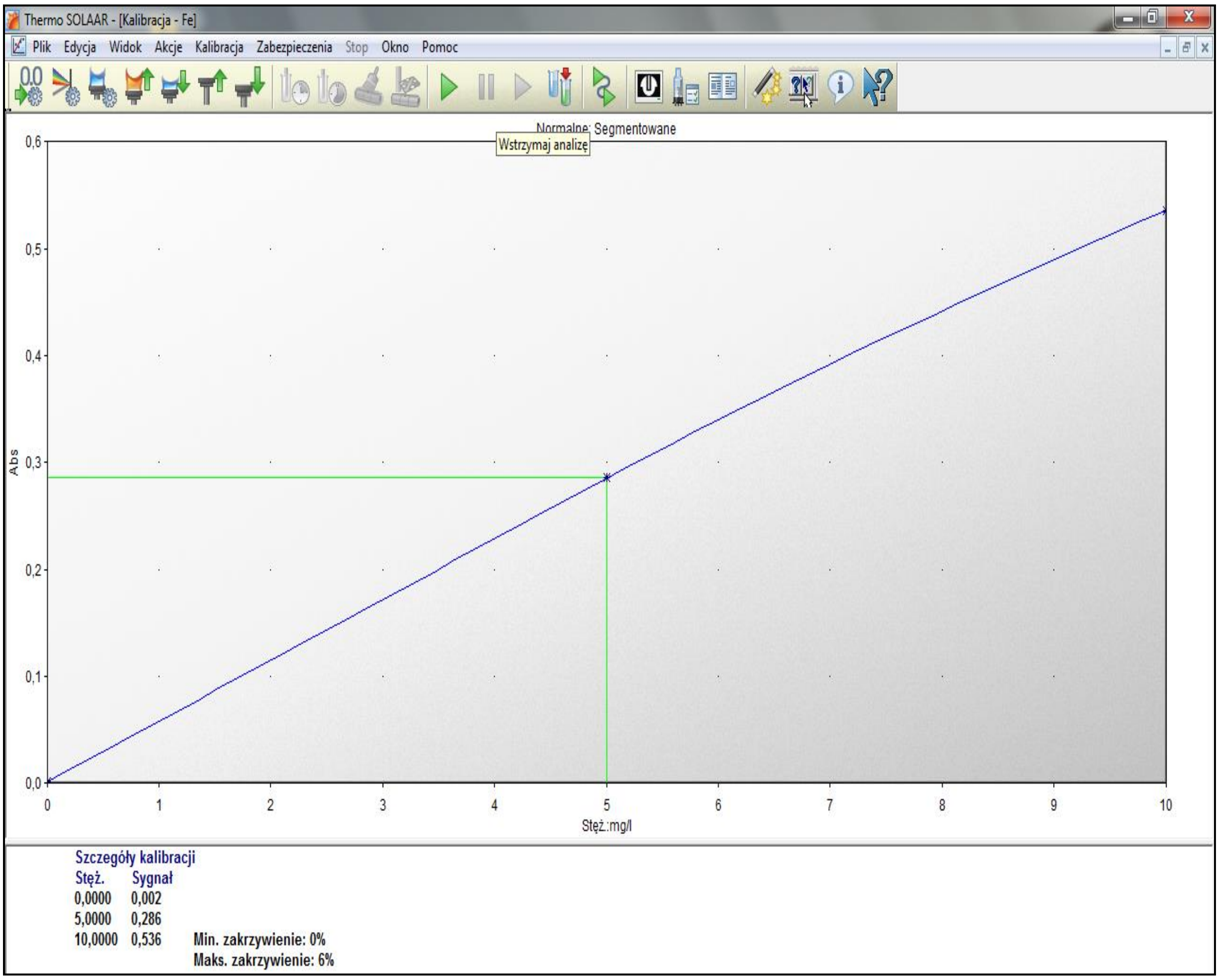

Fig. S5. The calibration curve used for the quantitative determination of iron (Fe). The curve was constructed using standards at concentrations of 0.0, 5.0, and 10.0 mg/L. The instrument software's internal validation showed that the curve's deviation from linearity ("zakrzywienie") was 0%, well within the acceptable limit of 6%. Legend: Kalibracja (Calibration), Abs (Absorbance), Stęż. (Concentration), Szczegóły kalibracji (Calibration Details), Sygnał (Signal / Absorbance), Zakrzywienie (Curvature / Deviation from linearity).

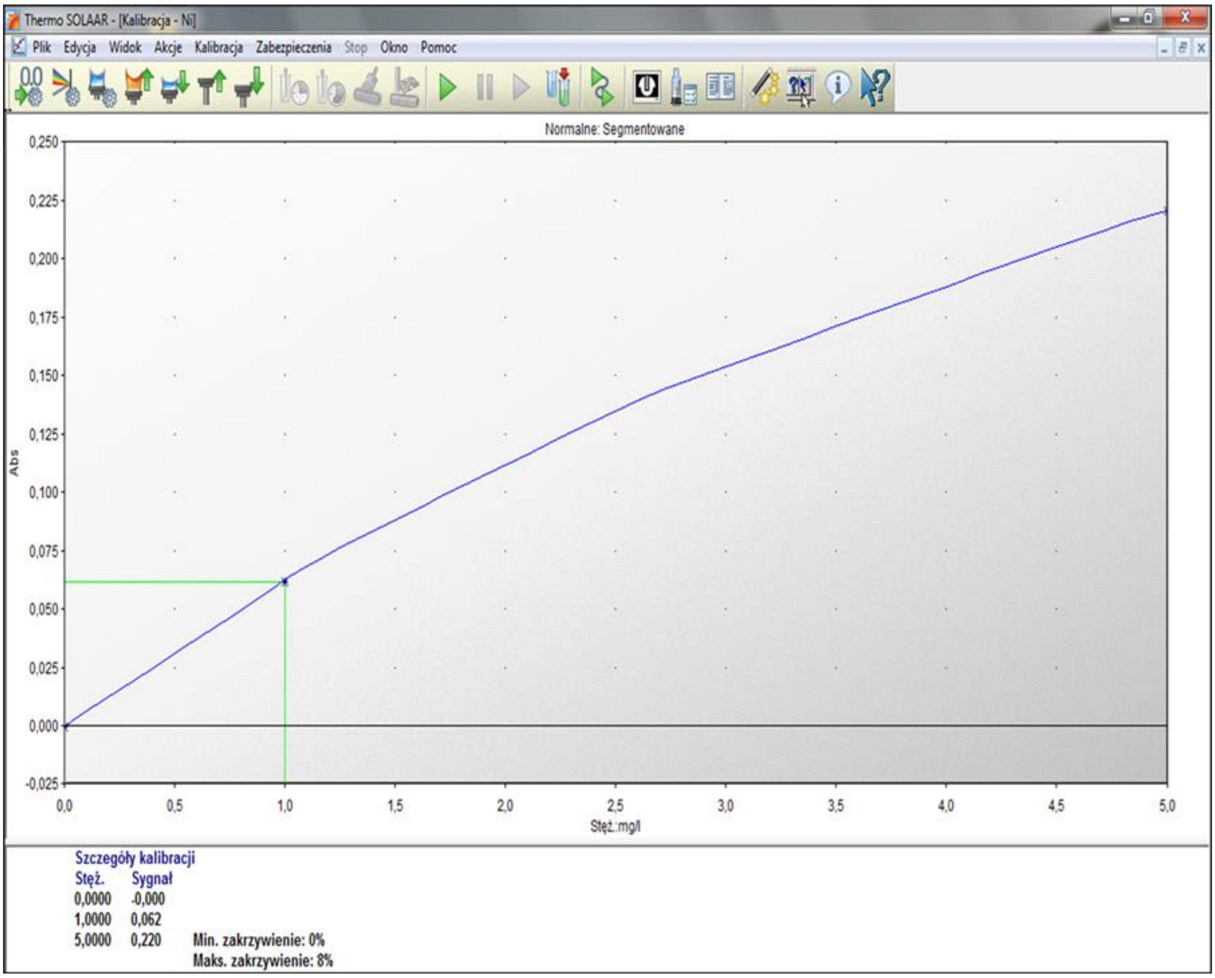

Fig. S6. The calibration curve used for the quantitative determination of nickel (Ni). The curve was constructed using standards at concentrations of 0.0, 1.0, and 5.0 mg/L. The instrument software's internal validation showed that the curve's deviation from linearity ("zakrzywienie") was 0%, which is well within the acceptable limit of 8%. Legend: Kalibracja (Calibration), Abs (Absorbance), Stęż. (Concentration), Szczegóły kalibracji (Calibration Details), Sygnał (Signal / Absorbance), Zakrzywienie (Curvature / Deviation from linearity).

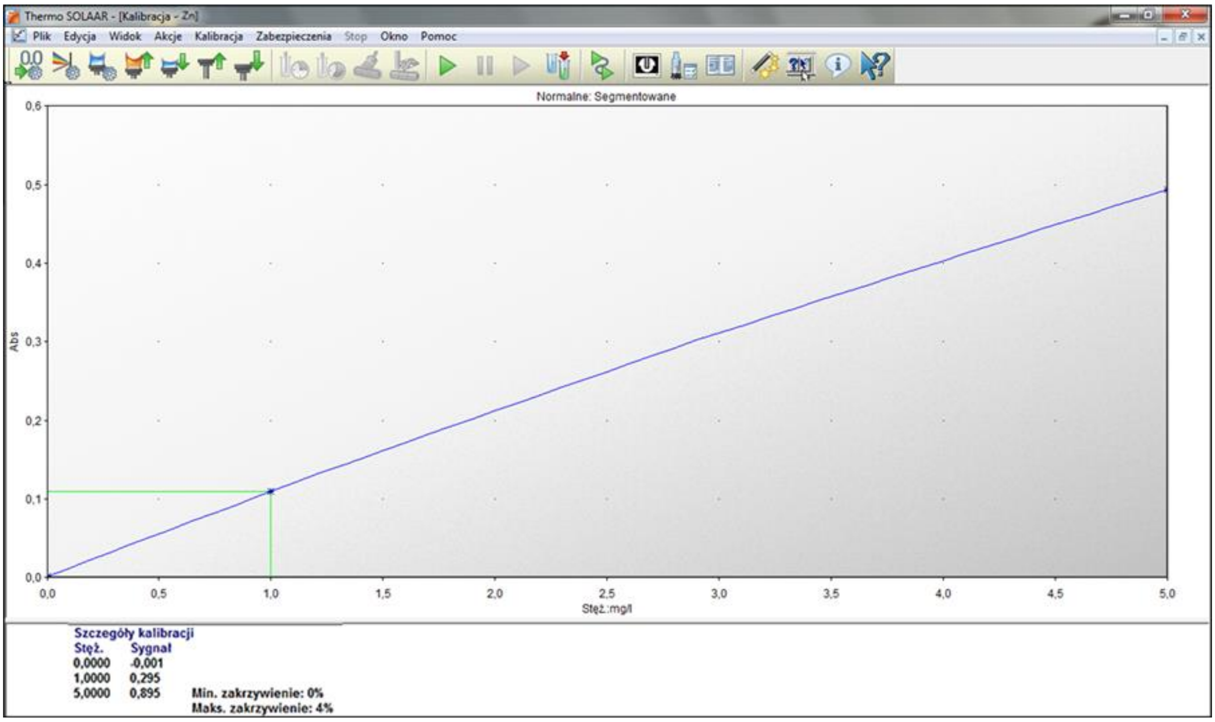

Fig. S7. The calibration curve used for the quantitative determination of zinc (Zn). The curve was constructed using standards at concentrations of 0.0, 1.0, and 5.0 mg/L. The instrument software's internal validation confirmed that the curve's deviation from linearity ("zakrzywienie") was 0%, which is well within the acceptable limit of 4%. Legend: Kalibracja (Calibration), Abs (Absorbance), Stęż. (Concentration), Szczegóły kalibracji (Calibration Details), Sygnał (Signal / Absorbance), Zakrzywienie (Curvature / Deviation from linearity).
